# ‘‘Time after time’’: A Quali-T method for assessing music's impact on well-being

**DOI:** 10.3402/qhw.v8i0.20611

**Published:** 2013-08-07

**Authors:** Tia DeNora

**Affiliations:** Department of Sociology, Philosophy and Anthropology, University of Exeter, Exeter, UK

**Keywords:** Music, ecological validity, temporal, well-being

## Abstract

This article considers the question of how to produce ecologically valid assessments of music's role as a health technology. To address this question, I consider critically some of the standard quantitative instruments used to assess well-being and quality of life. I suggest that these instruments do not lend themselves well to the production of ecologically valid assessments and understandings for two reasons: (1) the process of data elicitation is removed from everyday meanings and practices and therefore risks producing data that is an artifact of the situation in which it is elicited (2) standard, quantitative instruments are not neutral but are rather discursive texts that are inevitably imbued with a politics of expertise and an image of the health care client. For these reasons, I suggest that we consider the question of how to develop ecologically valid, client-centered assessment measures. To that end, I introduce a third critique of the standard quantitative instruments, namely that they are associated with, and promote, an ontology of wellness/illness that downplays the temporally variable and situationally emergent nature of both wellness/illness and musical interventions themselves. As an alternative mode of assessment, I suggest that we reconsider the value of singular case studies and I describe a set of principles that can assist researchers to produce ecologically valid assessments. To this end I introduce the concept of the musical event as a more ecologically valid means for illuminating the specific mechanisms by which music aids well-being. I suggest that the case study approach is temporally sensitive, that it lends itself to an emergent ontology of wellness/illness, and that it is client-centered (and can also be user-led).

Researchers in the area of music and health share, or often speak of sharing, a common commitment to the idea that music “helps” (MacDonald, Kreutz, & Mitchell, 2012; Ruud, 2010; Stige, Ansdell, Elefant, & Pavlicevic, 2010). For some, this commitment is linked to the idea that music might be available “on prescription” (Arts & Health Southwest, n.d.; Carlowe, 2011; Walker & Boyce-Tilman, 2002)—that music is clinically applicable, a therapeutic medium and thus not so dissimilar (in policy and procedural terms) from pharmaceutical interventions. For others, music is depicted as an everyday medium or cultural practice that can be integrated into the routine care and monitoring of the self and situation, linked to the regulation of mood, energy levels, and other symptoms, such as chronic pain, distress, and insomnia (Batt-Rawden, 2010; DeNora, 2000; Skånland, 2011).

But whether or not music is conceptualized overtly as a therapeutic intervention (as opposed to an everyday activity), many researchers working in the area of music, health, and well-being have responded, or felt the need to respond, to calls for evidence-based medicine (EBM) and evidence-based practice (EBP) (Bradt, 2008; Sabatella, 2004). In great part, the attention to evidence, and to music's effectiveness in or as an adjunct to health care, has employed quantitative research methods (so-called “hard” data) that are deemed able to measure music's impact in “reliable” and “objective” ways. Quantitative methods have, moreover, increasingly been linked to what is termed the, “gold standard” in EBM/EBP, the randomized control trial, or RCT, albeit not without some controversy (Slade & Priebe, 2001). Correspondingly, qualitative methods, and in particular case studies and user-led or user-centered designs, are often positioned at the bottom of the evidence-hierarchy (DeNora, 2007; Edwards, 2004; Wigram & Gold, 2012). And yet, in relation to music, community music interventions, and music therapy, the case for quantitative assessment remains open to debate. This article enters that debate from the point of view that, as one commentator put it, “… the question we need to consider is not *whether* to engage in EBP but *how* to engage in it” (Bradt, 2008). I shall suggest that this “how” entails a critical reappraisal of case study, person-centered approaches, and so this article poses the following question: how useful are quantitative instruments (in particular diagnostic survey questionnaires) for assessing music's impact as a health technology? I address this question through two key tasks. The first task is to critically consider some of the standard instruments used to measure music's impact on well-being. The second task is to propose an alternative mode of assessment that re-asserts the value of the singular case study.

## To measure health

Many aspects of music's role in relation to health and illness have been subject to measurement, RCT, and systematic review. These aspects include anxiety, schizophrenia and depression, autism, dementia, and pain perception. In all of these cases, the aim has been to employ “rigorous” (valid and reliable) methods to assess the regularity and extent to which music can reduce symptoms.

For this task, a range of quantitative measurement instruments are typically employed, most of which are generic, internationally standardized, highly used and highly regarded. These instruments take the form of self-assessment tools and observer-rated, clinical tools. Two prominent examples of the former is the *Hospital Anxiety and Depression Scale* (HADS; Zigmond & Snaith, 1983) with questions such as (“I feel tense or wound up” and “I feel cheerful”—“Yes definitely, Yes sometimes, No, not much, No, Not at all”) and the *SF-36 Quality of Life Survey* (Ware, 2000) with overview questions about self-perceived health such as, “*Compared to one year ago*, how would you rate your health in general *now*?—much better now than one year ago/Somewhat better now than one year ago/About the same as one year ago/Somewhat worse now than one year ago/Much worse now than one year ago” (Medical Outcomes Trust, 2006).

### Ecological validity and the meaning of the measure

While hailed for their reliability and increasingly employed as standard assessment devices, these instruments have also been subject to substantial critiquing. The starting point for this critique is the often-overlooked question of how *ecologically valid* (Cicourel, 1982) these measuring devices are and can be.

The critique of low ecological validity begins with the notion that assessment tools are themselves activities in the world and as such their administration and use occupies time and constitutes a situation in its own right. That situation might involve engagement with a trained assessor or might consist of the respondent (client, patient, subject, person) engaging with and completing a survey questionnaire. In either case, measurement takes place in a form of situation outside the context of naturally occurring everyday life and is linked to the situation of testing (sometimes literally, sometimes conceptually, a “white room” [lab or clinical/professional setting]). As an activity, the task of being measured or engaging in self-assessment via a questionnaire involves setting time aside (outside the daily flux) so as to complete the task, with or without the assistance of an interviewer. That task in turn involves formal assessment (judgments, estimates, cataloguing) made in the here and now of the assessment situation. To the extent that the assessment situation differs from everyday life as it otherwise transpires, there is the danger that the results of assessment may not be “ecologically valid” (Gobo, 2008; Marradi, 1990). Thus, the question arises:

Whether data gathered under controlled conditions are commensurate with routine problem solving and language use in natural settings. (Cicourel, 1996, p. 221; see also Saferstein 2010)

This question in turn raises issues of how to collect and analyse data so as to produce ecologically valid accounts of how music may promote wellness and health and for this, I suggest in what follows, that we can benefit by thinking critically about the tools that we use when engaging in EBP.

With Cicourel's thought in mind, the situation of responding to a survey can be considered as a type of everyday problem-solving situation, though not one that occurs under “routine” conditions. So, for example, it may involve asking the respondent to use non-indigenous terminology or categories of experience outside the normal bounds of how one is otherwise aware of and talks about one's situation (e.g., one might not normally “think about” one's experience in periods of time such as “the last month”). Thus, while instruments such as the HADS and SF-36 are discussed within the various literatures as highly reliable (i.e., measurements are replicable and consistent on repeat trials), and while they have been developed so as to be coached in “non-leading” language, the question arises whether they are actually measuring something that corresponds to “real-world” practice and experience. Are such measures valid proxies for that experience or are they ultimately measuring *literally* the answers given on the test occasion? How we gather ourselves and present ourselves to the “other” in a test situation is, in other words, often mediated by the test situation; it runs the risk of exhibiting what Cicourel speaks of as “white room effects”—responses that are produced in, because of, and appropriate to, the test situation.

In addition to “white room effects” and perhaps especially in relation to attempts to assess the type of “independent” variables that involve music as social practice (meaningful and fuzzily bounded interventions such as listening to music, participating in a choir or ensemble, music therapy), de-contextualized quantitative assessments run a second and related risk. As the object—music is (unlike drugs or some clinical techniques) not a stand-alone medium, fully standardized and “the same” from occasion to occasion (music's meanings and semiotic force is emergent and context-linked), there is the additional risk of confounding the experimental design due to “intervening variables” (i.e., loss of “control”). So, for example, when the double blind is broken or when research subjects believe (founded or not) that they are part of the experimental/treatment group, their measured rates of positive improvement rise in ways that may be attributed to the “placebo effect” (Kirsch, 2010). So too, if subjects believe they are receiving attention that goes beyond “treatment as normal” (the so-called Hawthorne effect [De Amici, Clersey, Ramajoli, Brustia, & Politi, 2000]) their test results may improve, irrespective of or in combination with whatever effects might be associated with the treatment variable. Thus, to repeat, administering a survey, and/or being part of an RCT of any kind in relation to health and well-being (even if in the “control” group) is a form of attention and may be linked to associated “Hawthorne effect” (Parson, 1974; Sekhri, 2011) such that it may be difficult to disambiguate effects or reported effects from procedures of elicitation and thus difficult to know how to account for any changes registered by assessment and measurement devices. For this reason, and in relation to pharmaceutical trials for depression medication, it is therefore not surprising that in many RCTs “improvement” is found in both groups, the control group and the treatment group and that there is often surprisingly little, even if statistically significant, difference between the improvement of each group (Kirsch, 2010). In short, the situation of treatment design and assessment may elicit responses from research “subjects” that are not commensurate with more routine forms of experience and action (Cicourel, 1996, p. 30; Cicourel 2007) and the actual involvement in the RCT may—especially when “fuzzier” variables are being tested and assessed (e.g., music and well-being rather than a medication and the degree to which blood can be made to clot) - itself elicit placebo or Hawthorne effects. Ecological validity thus highlights an important question: just how appropriate are quantitative instruments, and indeed the RCT, as a means for evaluating and testing the interrelationship between music and health?

There is another, related, problem: quantitative methods such as surveys completed outside of the context of daily routines offer retrospective perspectives on health and well-being. As such, and linked to the problem of ecological validity, retrospective accounts are, themselves, situated and thus subject to distortion (Kwon, Clarke, & Wodak, 2009). They are often elicited by asking respondents to engage in recall, but from within a time-boxed situation and without the benefit of contextualizing memory prompts such as, for example, video real-time data using techniques of playback to elicit subjective accounts [see Ruhleder & Jordan, 1997; Tudhope, Beynon-Davies, & MacKay, 2000], or diary data [i.e., a form of “recall” that was produced closer to the actual time of events recalled and without the help or hindrance of others’ recollections], or other prompts elicited from within an in-depth, ethnographic interview.

Thus, as with all survey questions, and many forms of (highly structured) face-to-face interview questions, the respondent is asked to take a stance and summarize his/her health or well-being status, over a time period of some length—3 months, a month, the last year. While respondents may give the same answers if tested repeatedly, those answers are considerably removed from the actual circumstances of the respondent's wellness situation (temporally and spatially). They therefore may not validly reflect the respondent's actual, moment-to-moment attitudes and capacities. In other words, a respondent may offer a discursive representation of his/her health/well-being (and survey instruments are, as I describe below, ripe for discourse analysis). But what she/he may state may bear little resemblance to actual temporal situations and moments of health and well-being: what we say, in other words, may not equate to what we do or what we experience. Rather, what we say outside actual contexts of our routines and activities may be linked to what we think we do, what we wish we did, or what we wish others to think we do and examples such as smoking, drinking or drug consumption, and eating patterns illustrate this point. (In other realms, e.g., athletic training, we measure the activity itself: we do not ask a swimmer whether he/she has gained speed. Rather, we ask them to race—in the usual way, to the best of their ability-clock their time and analyse their stroke.)

### Singularity and the issue of qualitative difference (part 1)

But there are additional problems that differentiate the condition of wellness/illness from other phenomenon and other behavioral conditions. For one, states of wellness/illness are *singular* and in at least two ways. First, we do not (typically) understand our own lives through statistics, and to ourselves, if not always to others, we are much more than contributors to statistical, aggregate data. For one thing, when we are suffering, we are less interested in the probability that some intervention might help, and more interested in whether and when it will help us, here, now and if it does not, why not. Illness, in other words, is experienced by individuals and thus it is, to those individuals, unique, a matter of the “here” and the “now”. To speak of this form of singularity is to entertain the idea that “real” reliability is unachievable through generic measures of assessment: “reliability cannot be achieved by the same procedures for all subjects, but only for each subject taken separately” (Marradi, quoting Cicourel, 1964, p. 80).

Second, what is often at stake for individuals is not the general trend (e.g., “my health has generally declined” or “my health is much better than it was last year”) but the ways in which a period of time takes shape from the qualitative events by which it is punctuated and structured (e.g., a string of memorable moments, a special event, a way of adapting and finding contentment, a way of eliding awareness of impediments and making do). Indeed, in this respect, health is not simply melded with well-being; it is well-being, a subjective and emergent state, a form of identity and, importantly, a particular resolution of aspirations, capacities for action, opportunities and self-perception in real-time and in situations. On this point, one website for users of the SF-36 and other quality of life instruments instructs physicians as follows:

If you are using this as a clinical tool it is good to discuss with your patient how they feel about their scores and what it means to them. As QoL is very personal, two patients who have the same scores on the SF-36 may actually feel very different about their QoL. (Framework for Measuring Impact, 2012)

It is at this point that researchers who aim to assess health technologies—whether drugs, or surgical techniques, or music—respond by saying that there is no way of producing compelling, robust data about effectiveness through a focus on the singular because singular forms of data, albeit their potential for ecological validity, share no common denominator. Instead of gaining the power that comes from large sample sets, we are left with seemingly incommensurable individual case studies. And yet, as Wigram and Gold consider:

The first challenge is the dichotomy of science and scientific fact versus subjective experience and individual preference. Wellbeing, as a concept, lends itself very strongly to an important but frequently unacknowledged aspect of EBP, that of ‘patient report’ – in other words the effect of a phenomenon or intervention on an individual that enhances or improves his or her own sense of wellbeing that may be unique to that individual and does not rely on scientific veracity for the effect to be accepted. (2012, p. 164)

In other words, the reality of individual experience highlights what Wigram and Gold describe as a dichotomy of scientific fact and subjective experience. The latter, within this dichotomous view, insofar as it cannot be measured, is often hailed as incommensurate with “science” because, so the reasoning goes, if each individual's experience is (potentially) unique, then generalizability (and hence music on prescription, following some generic algorithm) is impossible. We are, within this purview, forced to choose between generalizability and ecological validity. If we opt for the latter, there is (it would seem) no case in favor of health policies of music promotion and—equally importantly—no possibility of elevating music's profile as a health technology on par with clinical medicine.

It is precisely this dichotomy, and its adjacent assumptions about how individual experience is anathema to scientific study, I shall now suggest, that requires investigation. How, then, can we produce or seek to produce ecologically valid forms of arts and health evaluation? To begin to answer this research question, we need to set the scene with a critical consideration of measurement as discursive practice and that is the aim of what follows. This critique begins with the idea that measurement is always imbued with the politics of expertise. It moves on to consider how the range of evidentiary modes can and should be enriched in ways that are arguably more appropriate to music therapy and health-musicking. It ends by querying conventional ontologies of health and illness. This questioning in turn paves the way for rethinking the value of case study methodology as a client-centerd, idiographic and, most importantly, ecologically focused form of assessment, one that is grounded in actual events in people's (clients’) lives, whatever forms those lives may take.

## The move toward client-centered measures

### The discourse of “neutrality”

First, no measurement device is culturally neutral. Consider the following questions from the SF 36: “In general, would you say your health is: Excellent, Very good, Good, Fair, Poor?” Or consider this question from the HADS (where the respondent is asked to choose a degree of agreement with statements such as: “I have lost interest in my appearance” or “I can enjoy a good book or radio or TV programme.” Or, in the Beck Depression Inventory (for use with people aged 18–80 years), “I get very little pleasure from the things I used to enjoy,” “I am less interested in sex than I used to be,” or, “I am much more irritable than usual.” Treated as discursive texts, the questionnaires can be seen to embed certain assumptions about the state of health/illness, ontologically. (I return to this point below.) But they also embed certain normative presumptions (in the examples just considered, ones that are ageist and perhaps consumerist) about what counts as healthy and well: focused on physical appearance, interested in sex and TV, peppy, and feeling as “healthy” as ever despite the passage of a year, despite perhaps complex antagonistic circumstances at work or at home, and as someone who would not be seen to benefit from a pharmacological intervention (pills for shyness, diet, depression, anxiety, sexual performance, or youthful appearance as in Hormone Replacement Therapy). It does not take a Foucauldian analysis of the medicalization of personhood (Rose, 1996) to highlight the ways in which the subject depicted in these texts is highly regulated!

For example, such a vision may fail to validate the experience of, let us say, hypothetically speaking, an elderly arthritic person who cannot run, has perhaps lost interest in sex or shopping, who is wrinkled, sagging, grey, and balding (men/women) or bewhiskered (women), who no longer enjoys things that they previously enjoyed (“before they became fully mature,” “can't be bothered to dress up, don't need any new clothes or new car or new sofa even though I could afford these things”) and who believes that watching the world from a front window, balcony, park bench or even hospital bed is by far more interesting (“sweeter”) than a novel or TV! Similarly, this hypothetical person may not feel “happy” (as we typically use the term) much of the time but instead may feel a (perhaps more judicious) bittersweet amalgam of emotions (sadness, sorrow, grief, joy, regret, guilt, satisfaction, amusement, curiosity, disenchantment, and so on), a mixture perhaps “appropriate” (but not, however, normatively prescribed—one might indeed be ageing “disgracefully” [sic]) to their level of life experience (what has passed, what is present, what might be to come) and their physical capacities. That person might very positively relish the sense of “well-being” (alone and/or with others)—however laden with pain, difficulty, or discomfort—and might in fact have an overall quality of life that (at its best, at “peak” times—see Aasgaard, 2002, pp. 203–208) “equals” (the need for commensuration is itself a problematic matter, hence the use of scare-quotes) that of younger and more active, energetic, and culturally engaged respondents. And yet this ineluctable (perhaps tacit) sense of “well-being” might not translate onto the survey schedule where they are asked if they have felt particularly “peppy” over the last month.

Continuing with the Foucauldian theme of Power/Knowledge (Foucault, 1980) is the theme of politics of expertise. By what means should diagnosis and health assessment proceed? According to which—and whose—criteria? To answer this question, I suggest, we need to take a step back or “down” from the measures encoded in instruments of the type I have so far discussed. We need, by contrast, to return to the “groundedness” of health/illness and well-being in daily life and to reconsider more indigenous or “folk” measures in all their singularity (Faulkner & Thomas, 2002; Kitwood, 1997) and in all of their mercurial, temporal variability (Charmaz, 1993; DeNora, 2012, 2013): to be able to get out of the house, to feel bold enough to speak up at a meeting, to reduce or dispense with pain medication, to lose or gain weight, to “manage” to attend a special event, to be creative, to laugh, cry, take care, or not to care, about one's physical appearance, to be able to pursue that which one wishes to pursue, within some set of limits, to feel the sun on one's shoulders after a cold and rainy week. Returning to these “real-life” examples simultaneously helps to recapture the important role of collectivities and cooperation—the role played in one's health/illness/well-being by others (their help, compassion, engagement, cooperation), and by environmental materials such as furniture, architecture and transport technologies. It also reminds us of the ways in which health and well-being are not the properties of individuals but are shared and produced between individuals and are thus matters that remind us of our collective responsibility for keeping each other well.

## Enriching the range of assessment modes

In a thoughtful essay on EBP, Abrams (2010) has suggested that the orthodoxy of the EMB hierarchy, whereby “objective” and “global” forms of evidence (statistical measures, controlled trials) can and should be broadened to include forms of evidence that focus upon the individual and subjective domain. Abrams therefore offers a two-by-two table of evidentiary formats classified according to whether the assessment is focused on the individual or the collective and whether it seeks “subjective” or “objective” data. Abrams’ essay highlights how the dominant paradigm and its positioning of the RCT and measurement as the “gold standard” does some violence to music therapy's unique properties as a health technology, namely, its role as creative, meaningful practice. Music is, in other words, much more than a tool or instrument, and as such is not merely a means or ancillary to the achievement of some abstract thing that we call “health” or “well-being.” To the contrary, music and musicking are ends in themselves and thus their effectiveness involves what is deemed “good” and “beautiful” as well as a “true” means to health-linked ends. For these reasons, Abrams’ essay adds weight to the call for assessing music in ecologically valid ways, and in ways that, as he puts it, “target processes and outcomes that are valuable (i.e., effective and/or meaningful) both from a disciplinary stance and from the patient's (client's) point of view” and that involve various levels of participation and collaboration with the patient (client's) (Abrams, 2010, p. 358).

Similarly, Pavlicevic and Ansdell have invoked Goethe's notion of “gentle empiricism” (Pavlicevic & Ansdell, 2010, p. 132) to suggest that music therapy and its distinctive qualitative tradition offers a robust counter to, as they put it, “replacing the phenomenon with abstractions (be these in words or numbers).” Instead of endorsing what from now on I will now call the “blunt instrument” of survey questionnaires, Pavlicevic and Ansdell describe four alternative forms of assessment. These are: (1) using “musical” engagement as a technique of close observation (focused on how the other responds to sonically organized intervention) while simultaneously using it as a way of establishing relational contact; (2) a music-centered approach that considers how music comes to be transferred or “gets into” people's experiential and social lives where it can do things for them (such as help to organize cognitive, affective, and social experience); (3) expert (researcher) observation drawn from idiographic research that uses close listening, indexing, re-listening, and describing to get at participant's understandings and voices; and (4) simply asking clients themselves (Abrams’ third quadrant—individual, subjective data). The case studies that Pavlicevic and Ansdell use to illustrate these strategies are compelling and especially well chosen since they include situations where survey questionnaires would be inappropriate (most strikingly in the case of the first example where the client was a comatose man in an intensive care unit). The point, in short, is that gentle empiricism not only reaches the parts that some of the blunter instruments are unable to reach, it also reveals the manifold ways in which music actually “helps” in situ and in ways that allow them to feed directly into theory and at a level of generality that does not lose or traduce the phenomenon (DeNora, 2003, p. 40). Needless to say, these points also apply to cases where people are gravely ill, living with different degrees of dementia and memory issues, and neuro- and learning-disabilities—people who are thought “able” to offer valid or reliable forms of self-assessment. Indeed, as I shall argue below, they may indeed be “able” in ways that we are “unable” to perceive, all the more reason to “go gently” and that is the aim of the second section of this article. First, however, it is necessary to consider the third form of challenge to orthodox hierarchies of EBP and EBM in relation to music: the very idea of what it means to speak of health, illness, and well-being and what “kind” of things these are.

### New “ontologies” of health/illness

Assessing health/illness and well-being in terms of opportunities for action (Ruud, 2010) and, I will add, experience, is simultaneously assessing communities of care and thus acknowledging that wellness and illness emerge in relation to figured grounds, to what environments afford and do not afford. From this purview, a different ontology of health/illness can be glimpsed, or rather an anti-ontology in the sense that it points to much more ambiguous conditions of health/illness as taking shape—emerging as momentary configurations in relation to ecologies of action. I am/am not mobility disabled, for example, according to how I fit into institutionalized systems of material culture and technology (Freund & Fischer, 1982; Freund, 2001), communicative cultures and patterns of work (Groce, 1978). Thus, I have no one, fixed or given health/illness condition but rather that condition is itself conditioned according to what my environment of others and things and conventions can afford. The term affordance is significant and I will return to it shortly; for now suffice it to say that it highlights the moral–economical character of all health–illness identities that take shape through what things outside individuals can and will permit. There is, in other words, a cost—moral, economic, political—to what we wish to figure as well-being.

This “ecological” perspective recontextualizes music (it is now not a health intervention but a cultural practice) and thus paves the way for thinking about music and what it does for well-being and health/illness as more than a utility. It suggests that we think more about both encounters with music and aspects of well-being *in the singular, and temporally*, as moments, time after time, coalescing—or not coalescing—into dense or lightly textured patterns of being, and of being ill within being well and well within being ill. It is here that the importance of “idiographic” methods of assessment comes to the fore since they are the only methods capable of registering the often-vital qualitative differences—differences both between people and the differences that “make a difference to” people. As Ansdell and Meehan (2009, p. 36) have observed:

[t]here is a long history within music therapy and its research literature of attending closely to the single case and arguing for single-case designs as viable research methods for both developing practice and for providing evidentiary material (Adridge, 2005). Whilst most cases in health care research are still told from the perspective of the therapist, increasingly service-users are asking that clinicians also take into account their own accounts of their experiences of illness and the unexpected health they often find within illness. (Carel, 2008)

### Singularity (part 2)

In summary, quantitative measures are blunt instruments because they offer only generalities; they do not illuminate the singular features of health as it is lived and experienced moment to moment and as moments of wellness/illness day-to-day, hour-to-hour. Survey measurement irons out the crumpled, manifold texture of health/illness experience as it is lived, as a mundane temporal and situated, emergent reality. It then rearranges that reality as a set of health indicators and general statistics seen from within the purview of an inevitably value-laden, possibly ageist, consumerist and certainly medicalized lens of what it means to speak of “health.” This lens may be inappropriate to the lived cultural experience of health care clients and thus the criteria associated with survey methods may do damage to folksonomies (folk classifications) and folk or ordinary lived experiences of health/illness. Equally, such measures are—as Abrams (2010), Ansdell and Meehan (2009), and Pavlicevic and Ansdell (2010) have described, inadequate for perceiving and documenting just how it is that music helps (Maratos, Crawford, & Procter, 2011). If we do not attempt to open up the black box of music's mechanisms, its “active ingredients” (which may not be entirely or even mostly dependent upon the actual musical stimuli per se) we are left with yet another blunt technology—the correlation, in this case between (on the one hand) an overly general conception of what music is and (on the other hand) an overly general conception of what well-being is and how to measure it. And instead of gaining insight into what music actually does, our focus is narrowed to two time frames—before and after the musical “intervention,” and two correspondingly, and seemingly static, health situations, before and after music. The middle time period (the phase of actual musical engagement and the phase where music “gets into” how people are and what people do) is sidelined. We are left, to quote the poet Edward Dorn (from a series of poems written while driving up the US West Coast Highway 101, from Southern California through Washington):

One O One, that great Zero/Resting eternally between parallels. (Dorn, 1978, p. 74)

If we truly wish to know whether music makes a difference, and if so how and when, then we need to consider actual musical experience itself and not merely music's purported, retrospective “effects.” Rather, we need to examine musicking in, as it were “the middle” of music, music-centered, in other words (Aigen, 2005). This missing middle phase (what happens in interaction with music and qualitatively what happens musically during that time?) is of critical importance because, and in contrast to the time it takes to swallow a pill, the time it takes to make and consume music is not only of greater duration but qualitatively different—it consists of a form of symbolic and esthetic interaction with music and other things.

So, for example, instead of measuring a statistical correlation between music and improved self-reports of health, and instead of seeking to assure ourselves that music, the so-called treatment variable is not contaminated by any number of other factors (such as the social effects of performance [of anything] or collective activity [musical or other]), we should be willing to explore this complex assemblage of practice and “contamination” (mutuality) qualitatively. Testing music is, in short, not the same as testing drugs because music's “content” is always in flux, not stable, linked to the ways it is heard, contexualized, and rendered. Music is not, as I have already observed, a firmly bounded object in the way that a dose of medication is. With the latter, at least, we have specified the substance, the dose, and the precise contents of that dose. With music, as discussed above, many parameters remain unknown, and the variable itself is, in fact, not a variable because, unlike a drug, it cannot be separated from many other behavioral and symbolic matters. (In the recent years, drugs themselves have been questioned in this way—suggesting that even for chemical substances there is a tangle of mind/culture/embodied effects [see Kirsch, 2010].) In attempting to measure music's effects, in other words, we are attempting to assess something that we have not been able to define or control: without examining the musical experience “from within” therefore it is impossible to gain a precise understanding of the, “but how” question.

How, then, can we have a qualitative method that is sensitive to specific moments, ecologically valid, and able to handle the singular but still be used as a general health assessment tool for testing what music does for health status? In the final section of this article, I describe a methodology that is oriented to these issues.

## Alternative modes of assessment

Developing a qualitative alternative should, I suggest, be governed by the following principles: first, it needs to be sensitive to the ways in which well-being/illness is temporal and situated, and, as a consequence of these features, an ecologically emergent reality. Depending upon what else is happening, available mediators and who is doing what, conditions of wellness/illness can be heightened, transformed, and modified. For example, music can replace the sense of despondency or the sensation of pain—temporarily, even in extremis.

It is important to recognize the ways in which assessing how music helps does not benefit from a mechanical or “billiard ball” model of how music (as a substance) “affects” a static condition (the language of the RCT and its dependent/independent variables), but rather assessment involves a focus on the mutual constitution of music’ powers and the situations and circumstances that if comes to affect (DeNora, 2000). This mutual constitution involves specific, local forms of attention (from the very intimate and micro-sensation of pain “in” the body to the specific ways in which music comes to matter to particular individuals [biographical associations] and groups [past practices, conventions, relations]). And it involves actual, and often highly specific, crafted practices (our listening postures or locations, the volume level, version of the work, embodied practices of playing and handling music, repertoire, and musical choices, the social relations in and around musical ensemble). To the extent that it involves social relations and settings, it also involves particular ecological configurations (e.g., listening to music with the lights off, listening to music while receiving a massage or drinking wine, making music in a particular location or with particular people). Thus, the reality of both music and its effects can be understood to take shape in relation to each other and to the context in which they occur. There is no such thing as “the same” music twice since the musical “object” is always a performed and relational assemblage (Cook, 2003) that includes the quality of its reception.

### Indigenous criteria

Second, we need to consider “indigenous” or “folk” criteria and health-classification systems as criteria of assessment and this move, which is a move away from more traditional EBP, is also, I believe, a move toward more valid forms of evidence. This means, as Wigram and Gold described (quoted above) as the individual's (or group's) “own sense of well-being that may be unique to that individual and does not rely on scientific veracity for the effect to be accepted” (2012, p. 164). So, for example, health/well-being/illness would not be assessed from an external point of view but from within the purview and horizons of the (often multiple) realities of lived experience/aspirations of clients/people (and, at times, others close to them). These criteria should conceptualize well-being in specific ways (i.e., not merely as a sense of feeling better writ large). That means operationalizing well-being, for example, as capacities for and abilities to, and—equally importantly—in relation to actual experiential realities as relevant to respondents in the here and now. So, for example, we might operationalize well-being as the ability to walk to the corner shop. But we might also operationalize it as a person's lack of concern with how they are no longer able to walk to the shop (i.e., that criterion is irrelevant to them). The point is that criteria should be elicited from within the environmental milieu, and not necessarily by researchers. They may be elicited or offered by clients themselves and may come to light in the course of ordinary conversation, through attempted forms of action, and object use (‘I wish I could lift this heavy frying pan’), from carefully conducted, perhaps repeated from in-depth interview or from observations made by those who are closest to clients and who may be better attuned to what it is that is sought (e.g., “mother just wishes she could get out more to do her own shopping” or “my wife wishes she would not feel so anxious about leaving the house” or “I just wish he would not be so silent and stare at the wall for so long”). So too, criteria can be identified through imaginative exercises (though this risks a loss of ecological validity), obliquely, through discussions of literary materials, through an inventory of the daily routine or through a catalogue of what makes a ‘‘good day’’ (e.g., a review of highs and lows of the previous day, week or month) or through versions of participatory design techniques such as shadowing, elicitation through playback of audio–video recordings and or cooperative prototyping of user tools and tasks as a way of divining user requirements on the principle that we cannot always formultate in words what we need or wish and that this knowledge takes shape at a tacit, practical level: it is often easier to elicit knowledge about what might be helpful by reacting agaist what is not helpful (Tudhope et al., 2000). (The focus on indigenous criteria does not dismiss absolutely the role of clinical criteria, or of criteria as defined by those other than the client her/himself. To the contrary, there may well be times when others wish to intervene, to persuade individuals that “there might be more to it and you might wish to seek professional help”—indeed, well-being is a collaborative phenomenon and our self-understandings take shape in relation to what others help us to see, constrain us to see).

Along with the attempt to set indigenous criteria, the actual processes of criteria determination need to be made more transparent—who identified the criteria, where, how and why. To make these suggestions in favour of indigenous criteria is by no means an idiosyncratic suggestion. Rather, it is related to an already acknowledged perspective—the focus on user-centered, and indeed user-led research design. As one commentator argued, more than a decade ago in the BJP, “[p]sychiatrists should attach as much importance to user-led research in the processes of clinical decision-making as they do to randomised controlled trials. This has implications for continuing professional development and the training of psychiatrists” (Faulkner & Thomas, 2002, p. 3).

### When music is the “treatment variable” how to design the “assessment instrument”?

How, then, to develop indigenous criteria for assessing music's contribution to well-being? The first answer is to work with what is already in place, in the course of clients’ everyday lives. So, if musical activity (listening, participation) is already part of the everyday life experience that activity can be explored in terms of how it functions and particular “musical events” can be highlighted for further exploration. The second answer is to examine music as it is introduced to clients’ everyday lives as when, for example, the respondent might join an existing or newly formed musical group, receive music therapy, begin to attend a concert or concerts, or begin to engage in personal listening. The nature of the provision is not, in any generic way, important (though as case studies accumulate patterns may emerge).

The techniques by which data are elicited may also vary. Key is that they will seek to follow the ways and degree to which, irrespective of whatever the musical activity was, music “got into” or afforded activity that in some way facilitated a “good time” during and after—by the respondent's own criteria of well-being, indigenously articulated. Research methods capable of investigating this question include diary data, ethnographic interviews and (strategically targeted) ethnographic observations (shadowing individuals; focused ethnographic investigation Gobo 308) and more bespoke methods such as the repeated interview and virtual music communities developed by Batt-Rawden (2010).

In all cases, the aim is to examine, through respondents’ own criteria and ecologically grounded definitions how actual occasions, episodes, events, or moments of well-being may be musically founded. This musical “founding” may arise directly from and within musical activity (as for example when the process and pleasure of singing a song temporarily substitutes a state of mind with a different focus), or indirectly (as for example when one remembers or anticipates musical engagement before and after it has taken place). If the link is indirect, it can be classed as what Aasgaard terms, a “spin-off” (2002, p. 204) or practice that offers, “a means of expanding the present life world” of clients (e.g., children who are forced to endure life in the hospital while being treated for cancer [Aasgaard, 2002, p. 203]) in ways that these clients perceive as positive. Similarly, spin-offs arise when music comes to be connected to, or facilitates, the achievement of client-led criteria (“it gets me out of the house,” “makes me forget”). Whether direct or indirect, the challenge is to capture and be able to document these “good musical moments” time after time and as, if, and when they accumulate into patterns that are hailed (by respondents themselves plus by others with whom they are associated in accountable ways) as “improvement.” Thus, the study of how and how much of a difference music can make, how much music can be seen to “help” is directly proportionate to the extent that people use music and believe that music does enable them to expand opportunities for action and experience (Ruud, 2008). We are now at a place where it is possible to embrace such otherwise “problematic” matters as the placebo effect, mind/matter (and culture) interaction, and the performativity of well-being as assets to the promotion of health and well-being rather than obstacles to their assessment.

### A Quali-T approach and the musical event

So far, the methodology advocated has been inclusive of a number of focused qualitative techniques. However, if we are to be able to monitor user-centered forms of well-being over time in relation to music, and if we wish to assess, from within each case study whether music is associated with particular advances in well-being, some form of “capture” device is required that will allow for the documentation of the interrelationship between music activity and concrete outcomes linked to well-being (indigenously defined). And since well-being is temporally constituted, it is important to use a method that situates this interrelationship in time, so as to illuminate the actual connections between musical acts and changes in how one is or what one does. For this task, I propose the schema of the musical event, developed in the context of considering music's more general “effects” in social life and in relation to action (DeNora, 2003). The musical event is a simple, conceptual device that allows us to follow and register how musical engagement is linked to change, from one moment, one time, to the next.

The musical event draws together three time phases, the first and third of which frame the second. Time 1 is *the past*. It includes anything that an individual or group associates with music prior to the present moment (Time 2). It may include personal associations and memories, tastes, and musical practices and skills. The past also includes impersonal, generic, and conventional associations between music, action, and reception such as the set of musical forms, genres, and styles (as understood by actors) and the prior collective, organizational or institutional histories of the use of these forms, genres, and styles.

The second time phase (Time 2) is *the present*, when people are performing, talking about, listening to, writing about music. What is paired with music and how, when, and where is this pairing done and with reference to what other things? So, for example, as described above, how might a song be paired with a particular stylistic rendering and/or how might it also be paired with talk about that rendering, or talk about the song?

Finally, the third time phase (Time 3) is *the future*. At some later time (for example, in talk about a musical occasion), something happens that can be shown to be linked, in some way, to the musical engagement.

This cycle repeats such that Time 3 becomes Time 1 “next time round.” What is then of interest to researchers seeking to assess music's connection and causal links to health promotion and well-being is the degree of transformation and actual change—movement toward aspects of living and experience associated with criteria of well-being.

The musical event schema is one that can be used to trace musical activity as it comes to be translated into psycho-social activity and vice versa.

So, for example, at Time 1, one might hear a song on the radio that one likes because of its peppy rhythm, and then, (Time 2) while walking to the shops, one might walk “in sync” to the rhythm of the tune. This song might then enter into a habit whereby the next time (Time 3) one walks to the shops one hums the song to oneself as a way of “getting moving” and so the song takes on new meaning as a walking song (for the next Time 1). The legacy of this musical engagement is the form of facilitated movement, the “getting moving,” exercise and perhaps easier time one has when walking to the shops. (That walking in turn comes to permeate the song and its associations, which in turn inflects the next “Time 1”.) Or, to take a different type of example, in Time 1, one might have memories of a song and its connection to someone one once knew, such that, at Time 2 when one hears the song on the radio one is reminded of that person so that, at Time 3, one goes back to a photograph album to look at old photos or one rings up that person after many years of not having been in touch (thus enhancing social connections). Or, a third, and more overtly therapeutic example and one that I have seen repeatedly in the work I have been conducting with Gary Ansdell (Ansdell & DeNora, 2012), one works on a particular song in one-to-one music therapy sessions such that it becomes a “favorite,” one performs that song, at Time 2, in a community music therapy session in front of others and, at Time 3, is able to talk about how one performed a solo (i.e., has some “nice news” to tell) and/or one feels a sense of accomplishment and heightened confidence such that, for the first time, one feels “strong enough” to go out to the shops on one's own, having previously felt afraid to engage in that kind of public venture.

In all of these examples, it is possible to follow the actual links between music and other things (actions, forms of embodiment, social ties), as they are made by those people themselves. The links, in ethnographic detail and accumulating over time, can be understood as the actual documentation of how music helps. This, in short, is what I mean by a Quali-T method—qualitative, temporally grounded, situated and client-centered, focused on how the actual, minute engagements with music, *time after time*, accumulate in ways that can result in significant forms of transformation and in the forms of change that we colloquially describe as “getting better.”

## Conclusion

The aim of this article has been to subject quantitative forms of assessment in relation to music and well-being to critique on the grounds that they are not ecologically valid, and to propose an alternative that re-asserts the value of the singular case study. The revaluing of case studies and qualitative methods of investigation and assessment does not of necessity exclude the use of quantitative measures and indeed, one could use the various scales that I have described as an adjunct to the Quali-T method. However, the scales would need to be contextualized (one might speak of this as bespoke calibration) within each case study if they are to be ecologically valid and if they are to avoid imposing possibly inappropriate values upon individual clients. Moreover, their role as active ingredients in their own right (cf the discussion above of placebo effects) would need to be acknowledged.

While some might suggest that using qualitative methods such as these are restricted to small samples (or unduly time consuming), consider that the more prominent RCTs on music and mental health in the past decade have had, respectively, sample sizes of 79 (Erkkilä, Punkanen, Phil, & Fachner, 2011) and 81 (Talwar et al., 2006). These sample sizes are not so large as to prohibit a qualitative and client-centered component (and at least in principle most music and health researchers advocate mixed methods anyway). The use of qualitative, case study and ethnographic methods is not necessarily more costly in terms of human hours required for administration, record keeping, data collection and analysis (Sibbald, 1998). And—if we are to involve clients in client-led forms of assessment, then, unlike RCTs, the method is economically sustainable—there is no need for large data sets and the singular case studies can accumulate in their own time and over time and since in all cases the criteria of assessment are indigenous and local.

In short, I have suggested that we consider the ways in which musical activity generates many types of affordances for well-being and sociability and that we examine this question through case-by-case studies so as to trace the ways that music enters into well-being (defined in client-centered ways and substantively, in terms of actual outcomes rather than general and abstract measures such as “Over the last month I felt …”). In this sense what music does in relation to health and well-being is not dissimilar to what music does in relation to forms of social agency and forms of consciousness/perception in other life realms where it has been documented as getting into action. A Quali-T method allows us to study the differences that music makes from “the inside,” by following music as it “leads” people into situations of well-being that do, and/or do not, accumulate over time. Time after time, musical engagement after engagement, change for the “better” is achieved through a series of socio-musical practices in the enclaves of everyday life and routine. We can afford to use this method and, I have suggested, we cannot afford not to use it if we seek ecologically valid accounts of how music helps.
